# 

**Published:** 1909-04-15

**Authors:** 


### CONTENTS

Event and Comment - - - - - - - - 173

"Watch Rochester—Give the Children a Chance,"
*By Mr. Horace Fletcher* 180

Dentistry and Preventive Hygiene, - *By Mr. Horace Fletcher* 193

Something More and Important About Dr. Taggart and the Dental Profession. - - *By Edmund Noyes, D.D.S.* 207

Indents - - - - - - - - - - - 213

Announcements - - - - - - - - - 223

